# The Genetic Variability of Present‐Day Bulgarians Captures Ancient and Recent Ancestral Contributions

**DOI:** 10.1002/ajpa.70037

**Published:** 2025-04-09

**Authors:** Stefania Sarno, Fedora Piccini, Paolo Abondio, Elisabetta Cilli, Elena M. Kuyumdjian, Nedko A. Dimitrov, Chavdar D. Dilov, Sara De Fanti, Graziella Ciani, Davide Gentilini, Alessio Boattini, Marco Sazzini, Davide Pettener, Donata Luiselli

**Affiliations:** ^1^ Laboratory of Molecular Anthropology & Centre for Genome Biology, Department of Biological, Geological and Environmental Sciences University of Bologna Bologna Italy; ^2^ Laboratory of Ancient DNA, Department of Cultural Heritage University of Bologna Ravenna Italy; ^3^ Bulgarian DNA Project FamilyTree DNA Sofia Bulgaria; ^4^ Central Balkan Mountains DNA Project FamilyTree DNA Varna Bulgaria; ^5^ IRCCS Istituto delle Scienze Neurologiche di Bologna Bologna Italy; ^6^ Bioinformatics and Statistical Genetics Unit, Istituto Auxologico Italiano IRCCS Milan Italy; ^7^ Medical Statistics and Genetic Epidemiology Unit, Department of Brain and Behavioral Sciences University of Pavia Pavia Italy; ^8^ Interdepartmental Centre Alma Mater Research Institute on Global Changes and Climate Change, University of Bologna Bologna Italy

**Keywords:** Balkan Peninsula, genetic heritage, genomic ancestry, migration processes, population dynamics

## Abstract

**Objectives:**

Thanks to its pivotal crossroad position, Bulgaria played a fundamental key role during all the migration processes that interested the continent through time. While the genetic variability of the country has been deeply investigated using uniparental markers, previous genome‐wide autosomal‐based surveys mainly consisted of wider‐range analyzes on Europe and the whole Balkan Peninsula. Here, we specifically focused on the Bulgarian population to recapitulate the main patterns of genomic variation and the major events that shaped the present‐day genetic landscape.

**Methods:**

A total of 112 samples from seven highly representative areas of present‐day Bulgaria were collected and genotyped for approximately 720 K genome‐wide SNPs, and integrated with previously generated genomic data from wide modern and ancient reference panels to explore fine‐scale relationship patterns and detail ancestral contributions.

**Results:**

In addition to the combination of ancient ancestries related to the early Mesolithic hunter‐gatherers, Neolithic farmers, and Bronze Age Steppe pastoralists, both haplotype‐based analyzes on modern populations and the comparisons with ancient genomes suggest the contribution of population processes that have occurred after the Roman rule and during the Medieval period in shaping the current Bulgarian genetic pool.

**Conclusions:**

Our results align with previous evidence highlighting the impact that some historical events may have had not only in contributing to the ethnical and socio‐cultural richness of present‐day populations, but also in participating in the formation of the current genomic landscape. By providing new data from modern highly‐representative samples, this study integrates further research to provide a comprehensive overview of the genetic history of Bulgaria.

## 1 Introduction

Present‐day Bulgaria, located in the south‐eastern part of the Balkan Peninsula, has historically represented a pivotal crossroad, connecting southern Europe on the west, the Near East on the east, central and eastern Europe on the north, and the Mediterranean on the south. Different types of evidence have largely indicated its significance since the first settlement of Europe during the Upper Paleolithic, as testified by one of the earliest human remains discovered at the Bacho Kiro Cave (Fewlass et al. 2020; Hublin et al. 2020). Furthermore, besides acting as an important refuge during the Last Glacial Maximum, the Balkan Peninsula has also been deeply involved in the spread of the Neolithic from the Middle East, that reached Eastern Europe by the end of the 6th millennium bce, as well as in the subsequent Post‐Neolithic expansions of the Metal Ages (Bailey 2000). Accordingly, the Neolithic culture of Karanovo, named after the homonymous Bulgarian village, is considered among the largest settlements in Eurasia, and the Chalcolithic necropolis of Varna, located in northeastern Bulgaria and dated to the mid‐5th millennium bce, testifies to the earliest known evidence of gold processing in the world (Mathieson et al. 2018; Penske et al. 2023). During the Bronze Age, the area of present‐day Bulgaria witnessed the formation of Thracian tribes (Fol 2008; Mihailov 2015), culminating in the establishment of significant kingdoms in the so‐called Thracian Golden Age (6th–3rd century bce). By the 2nd century bce, the area started being incorporated into the Roman Empire in a process completed by 46 ce (Iliev 2015). Due to the increased migration, urbanization, and integration into the broader economic and cultural life of the Empire, the region experienced deep ethnic and territorial transformations under Roman rule. Furthermore, with the Migration Period and after the fall of the Western Roman Empire, other populations also moved and settled in the Balkans (Angelov 1978; Stanev 2012). Among them, the Slavic‐associated settlement starting from the 6th century ce (Koder 2020), profoundly influenced both the cultural and the demographic landscape of the region. In the late 7th century ce, the Bulgars or Proto‐Bulgarians, a Turkic‐speaking semi‐nomadic people, pushed by Khazars, started to migrate out of the southern Steppes and the northern Caucasus region where Old Great Bulgaria had been founded around 632 ce, covering parts of what is today southern Ukraine and Southwestern Russia. One group of Bulgars, led by Khan Asparuh, migrated westwards and eventually settled into the northeastern Balkans (Angelov 1978; Dimitrov 1987; Rashev 2007) where they established the First Bulgarian Empire in 681 ce. The empire, centered on what is today Bulgaria, grew significantly in power and territory, becoming a major political and cultural center of the Balkans. During this time, the Bulgars began to integrate with the local Slavic populations, adopting Slavic language and culture. The First Bulgarian Empire collapsed after the Byzantine conquest in 1018. The Byzantine rule in Bulgaria ended in 1185 with the restoration of the medieval Bulgarian State and the foundation of the Second Bulgarian Empire, while 1453 marks the beginning of almost five centuries of Ottoman rule (Matanov 2016, 2022).

Reflecting its complex population history, the genetic landscape of current Bulgarians is the product of multiple migrations and interactions that have influenced the genetic diversity of the region over time. Previous studies, based on uniparental markers, that originally focused on the genetic variability of present‐day Bulgarians, largely indicated that they exhibit a haplogroup composition typical of the West Eurasian populations, tracing their ancestry to lineages of the Southeastern European gene pool and showing particular affinity to the other neighboring groups from Greece and the Balkans (Calafell et al. 1996; Karachanak et al. 2012, 2013; Karachanak‐Yankova et al. 2015; Kushniarevich et al. 2015; Modi et al. 2019). Accordingly, later studies that analyzed modern patterns of genomic variation using autosomal genome‐wide data agreed in revealing a relative genetic uniformity among the current populations of the Balkan Peninsula, locating them between the genetic variability of Eastern Europe and the Mediterranean, and supporting the scenario that the Balkans served as a crucial crossroad between the Middle East and Europe, representing one of the major routes of ancient and recent gene flows and admixture (Kovacevic et al. 2014).

Looking at the ancient contributions to the genetic pool of modern populations, more recent studies based on ancient DNA (aDNA) provided important insights, specifically suggesting that present‐day Europeans overall descend from three main ancestral sources, reflecting the genetic heritage of the Mesolithic Western European Hunter‐Gatherers (WHG) who largely replaced pre‐existing European populations after the Last Glacial Maximum, and the major impacts of farmers expansion from Anatolia during the Early Neolithic, and of the arrival of Yamnaya Steppe pastoralists during the Bronze Age (Lazaridis et al. 2014). While the Yamnaya‐related ancestry is generally higher in Northern and Eastern Europeans, modern Southern European populations were shown to exhibit relatively higher amounts of Anatolian Neolithic contribution. Accordingly, a three‐way mixture model considering WHG, Anatolian Neolithic, and Yamnaya‐related ancestries showed that present‐day populations of the Balkan Peninsula predominantly descend from Anatolian farmers, with the remaining part of their ancestry being assigned mostly to Yamnaya‐Steppe groups, and lastly to WHG (Haak et al. 2015). Subsequent aDNA‐based studies focusing specifically on the genetic history of the Balkans during the first millennium CE further showed the impact on the genetic ancestry of present‐day Balkan people of two other major events that took place after the end of the Iron Age. A large‐scale influx of people whose genetic profile matched that of contemporary Western Anatolian and Northern Levantine populations was detected during the Roman Period. Furthermore, a significant contribution from individuals with Eastern European‐related ancestry genetically similar to modern Slavic‐speaking populations appeared all across the Balkan region after 700 ce (Olalde et al. 2023).

In this framework, with the aim to provide an integrated perspective onto the genetic history of Bulgaria, in this study we collected and genotyped approximately 100 samples from seven highly representative areas of present‐day Bulgaria, and explored their fine‐scaled genetic relationships with both modern and ancient populations in order to detail the ancestral contributions that have shaped the genetic landscape of the region through time.

## 2 Materials and Methods

### 2.1 Samples Collection and Genotyping

Saliva samples analyzed in the present study were collected from a total of 112 individuals coming from seven different sub‐regions of Bulgaria (Figure S1, Table S1), namely: North‐western Bulgaria (NW), Central‐western Bulgaria (CW), Rhodope Mountains (RD), Thracian valley (TR), Central Bulgaria (CB), and two Bulgarian ethnographic groups—Vayatsi (VY) and Kapantsi (KP). The Vayatsi originally inhabited a few villages in the Eastern Balkan Mountain Region to the south of Varna city, namely Golitsa, Kozichino, Dobrovan and Asparuhovo. They have a distinct dialect, which is one of the oldest in Bulgaria, as well as their own specific costumes and folklore. The Kapantsi people are instead native of north‐eastern Bulgaria, mainly found around the city of Razgrad. While they are not very different from other Bulgarian ethnographic groups of the region in their traditions, costumes or dialect, the name of Kapantsi have generally brought some prestige to the people of that group. Both Kapantsi and Vayatsi indeed consider themselves as the direct descendants of the old Bulgars who came to the Balkans in the 7th century. Sample affiliation to a specific group/region of origin was surveyed over at least three generations according to the “grandparent sampling criterion”, and only individuals whose both parents and four grandparents were born in the same region of origin were selected for the analysis.

All the procedures concerning this population genetics study were approved by the Bioethics Committee of the University of Bologna on April 8, 2013 (within the framework of the ERC‐2011‐AdG 295,733 project) and the study was designed and conducted in accordance with the ethical principles for research involving human subjects stated by the WMA Declaration of Helsinki. The collection of samples was performed in agreement with and thanks to the engagement and participation of local members of the study population, and all the participants signed a written informed consent to sample collection and data treatment, after being fully informed by their local representatives about the purposes and the methods of the study. The sample analyzed in this study represents a fraction of the populations living in the regions of Bulgaria, and so it is only partially representative of the complex demographic and cultural history of these regions and of their inhabitants' ancestors.

Saliva samples were collected from each donor with the Oragene‐DNA Self Collection Kit OG500 (DNA Genotek, Ottawa, Ontario, Canada). Genomic DNA was extracted with the prepIT‐L2P protocol according to the manufacturer's recommendations, and quantified with the Qubit dsDNA Broad‐Range Assay Kit (Invitrogen Life Technologies, Carlsbad, CA, USA). Samples with good quality and quantity of DNA were genotyped for 713,599 single nucleotide polymorphisms (SNPs) distributed along the whole genome, using the HumanOmniExpress‐24 v 1.2 BeadChip (Illumina, San Diego, CA, USA). Successful results were obtained for 79 out of the 112 collected samples.

### 2.2 Quality Filtering and Data Curation

Obtained genotype data for the Bulgarian samples were filtered using the PLINK 1.9 software (Chang et al. 2015) by excluding all markers with more than 2% of missing data, variants showing significant deviations from Hardy–Weinberg equilibrium after Bonferroni correction for multiple testing, and SNPs with a minor allele frequency (MAF) lower than 1%. Additionally, individuals with a missing call rate exceeding 8% and one individual from each pair of samples showing a PI‐HAT value higher than 0.125 (indicating third‐degree of relatedness or higher) were removed from the dataset. Following these filtering steps, a final dataset of 38 Bulgarians typed for 378,648 SNPs was retained for downstream analyzes.

In order to perform population genomic analyzes aimed at exploring the geographic and temporal relationships of analyzed population samples with the ancient and recent patterns of genomic variation observed in Europe and the Mediterranean, the Bulgarian samples were then merged with two additional reference sets of data extracted from the literature.

A “modern” reference panel was made up of publicly available data from present‐day European, Middle Eastern, and Caucasian populations typed on various Illumina arrays (Behar et al. 2010, 2013; Busby et al. 2015; Kovacevic et al. 2014; Kushniarevich et al. 2015; Li et al. 2008; Raghavan et al. 2014; Tambets et al. 2018; Tamm et al. 2019; Yunusbayev et al. 2012, 2015). The same quality checks (QC) described above for the Bulgarian samples were also applied to the reference dataset. After merging, a modern extended panel encompassing a total of 1571 individuals from 84 populations (Table S2) genotyped for a common set of 144,366 SNPs (43,883 after pruning for *‐‐indep‐pairwise 50 10 0.1*) was obtained for subsequent analyzes.

This modern dataset was then merged with genome‐wide data from a set of 522 relevant ancient individuals from previous publications (Lazaridis et al. 2022; Mallick et al. 2024; Olalde et al. 2023) typed for 1,233,013 sites along the genome (Table S3). The merging process between ancient samples and the modern extended dataset resulted in a set of 140,054 common SNPs (105,084 after pruning for *‐‐indep‐pairwise 200 25 0.4*). All ancient samples that were previously annotated as low‐coverage, contaminated, outliers, or related samples were excluded from the comparisons.

### 2.3 Population Genetics Analyzes

A Principal Component Analysis (PCA) was performed on the populations of the modern extended dataset using the *smartpca* function of the EIGENSOFT package (Patterson et al. 2006). Subsequently, ancient samples were projected onto the PCA space obtained from the present‐day genomic variability, using the “*lsqproject = YES*” option.

Inferences of ancestry proportions for the modern population groups were estimated with the software ADMIXTURE (Alexander et al. 2009), by testing from *K* = 2 to 10 hypothetical ancestral components. For each given *K* value, 10 independent runs were performed and the cross‐validation error (CV) was calculated in order to determine the most plausible number of putative genetic components consistent with the data. Finally, only the run with the highest log‐likelihood value at each *K* was used for the final plot.

To visualize spatial patterns of genetic variation and population structure across the Balkans and surrounding regions, we estimated Effective Migration Surfaces using the *bed2diffs* and *runeems* tools of the EEMS package (Petkova et al. 2016), based on the average pairwise distances between populations. EEMS analyzes were performed assuming 500 demes across the considered range, by running EEMS for 3 million iterations with a burn‐in of 2 million iterations and a thinning iteration of 9999. The resulting outputs were visualized by using the *rEEMSPlots* package (Petkova et al. 2016).

The patterns of genetic relatedness that emerged among present‐day populations were further explored with the haplotype‐based method implemented in the CHROMOPAINTER/FineSTRUCTURE approach (Lawson et al. 2012). The modern samples of the unpruned extended dataset were phased with the software SHAPEIT (Delaneau et al. 2013) according to default parameter settings and using the HapMap phase3 recombination maps. The mutation/emission and the switch rate parameters were calculated by running CHROMOPAINTER on a subset of four chromosomes (4, 10, 15, 22) with 10 iteration steps of the Expectation–Maximization (E–M) algorithm. The estimated parameters were then averaged across individuals and by chromosomes, and used for a second run of CHROMOPAINTER on all the 22 autosomes. The obtained co‐ancestry matrix was submitted to the fineSTRUCTURE clustering algorithm, by initially setting 3,000,000 “burn‐in” iterations, and 2,000,000 MCMC runs sampled every 10,000 iterations. A second run of fineSTRUCTURE with 1,000,000 “burn‐in” iterations and 1,000,000 additional hill‐climbing steps was lastly performed to reach the final configuration tree in which modern individuals were hierarchically assembled into 67 genetic‐based clusters.

In order to identify and date admixture events that may have influenced the genetic history of the analyzed populations, we then used the software GLOBETROTTER (Hellenthal et al. 2014) based on the modern population clusters identified by ChromoPainter/fineSTRUCTURE. Specifically, GLOBETROTTER was run using the inferred *BULGARIA_MACEDONIA_ROMANIA* cluster, which encompasses the analyzed Bulgarian samples, as the target population, while all other European, Middle Eastern, and Caucasian clusters were used as surrogate population groups. However, since geographically proximate populations tend to share more recent ancestry, potentially masking more distant genetic contacts (Hellenthal et al. 2014; Tamm et al. 2019), a second analysis of GLOBETROTTER was performed excluding the clusters from the same South Eastern‐European/Balkan macro‐group as possible sources of the target Bulgarian/Eastern‐Balkan cluster. For each analysis, GLOBETROTTER was initially run using the NULL option with 100 bootstrap resamples in order to estimate *p*‐values for the admixture evidence. Then, a second run was performed using the non‐NULL inference to properly characterize the admixture events by implementing 100 bootstrap resampling to infer confidence intervals around the obtained estimates. The consistency of the results between the two GLOBETROTTER runs was also assessed, and a generation time of 30 years was considered for the conversion.

To infer the deep ancestral composition of ancient and present‐day Bulgarian populations, we exploited the approach implemented in the *qpAdmix* software (Harney et al. 2021; Patterson et al. 2012) by considering acceptable models with *p*‐value > 0.01 and for which the estimated proportions of admixture were non‐negative.

We began by testing a five‐way mixture model including distal ancestries that were shown to account for the vast majority of ancestry in European and Near Eastern populations. Specifically, as reported previously (Olalde et al. 2023) we used Caucasus hunter‐gatherers (CHG), Eastern European hunter‐gatherers (EHG), Serbia and Romania Iron Gates hunter‐gatherers (Iron_Gates_HG), Neolithic individuals from the Levant (Levant_N) and Neolithic individuals from Anatolia (Anatolia_N) as putative “source” groups (“*Left*” populations). Accordingly, as (“*Right*”) outgroups, we considered a set of ancient populations (Olalde et al. 2023) which includes: (i) ancient African hunter‐gatherers with no evidence of Eurasian admixture (OldAfrica) as basal outgroup; (ii) ancient North African (Morocco_Iberomaurusian) and East Asian (Mongolia_EIA) individuals to account for eventual East Asian‐related and North African‐related ancestries; (iii) six groups/individuals exhibiting differential relationships with the five source populations, used to effectively distinguish them: respectively, Neolithic individuals from Iran (Iran_N), Natufian individuals from Israel (Israel_Natufian), the 24,000‐year‐old Mal'ta individual from south‐central Siberia (MA1), an Epipaleolithic individual from Anatolia (Anatolia_Epipaleolithic), the Western European hunter‐gatherers (WHG), and Mesopotamian Pre‐Pottery Neolithic individuals (Mesopotamia_PPNA).

Finally, to focus more specifically on the more recent events that shaped the genetic ancestry of modern populations, we modeled the ancestry of present‐day Bulgarians by using a more proximate model considering source populations temporally and geographically closer to Bulgarian individuals. Accordingly, as recently reported by Olalde et al. (2023), we considered a four‐way mixture model including the following source (“*Left*”) populations: local Bulgarian Iron Age individuals (Bulgaria_IA), Balkan individuals from Serbia and Croatia deriving entirely their ancestry from Roman/Byzantine populations of Western Anatolia (CroatiaSerbia_RomanAnatolian), Central/Eastern European Early Medieval individuals clustering with present‐day Slavic‐speaking populations (CEE_EarlyMedieval), and Western Anatolia individuals of the Ottoman period (Ottoman_Anatolian). The corresponding set of (“*Right*”) outgroups was composed of: OldAfrica, EHG, Iron_Gates_HG, Anatolia_N, Iran_N, individuals from the Bronze Age Steppe Yamnaya (Steppe_BA), Iron Age individuals from Iberia (Iberia_IA), Minoans from present‐day Greece (Greece_Minoan), Middle‐Late Bronze Age individuals from Croatia and Iron Age individuals from Slovenia (CroatiaMLBA_SloveniaIA), Roman and Byzantine individuals from Southeast Turkey (SoutheastTurkey_Byzantine), Bronze Age individuals from Latvia and Lithuania (Baltic_BA), Iron Age individuals from the Central and Eastern Steppe (Steppe_IA), and Middle Bronze Age–Iron Age individuals from the Netherlands (Netherlands_MBA_IA).

## 3 Results

### 3.1 Present‐Day Patterns of Genetic Structuring and Admixture

A preliminary investigation on the patterns of genetic variation among the newly genotyped Bulgarian individuals was initially performed in order to assess the presence of potential population sub‐structure among the collected individuals. While being aware that the relatively low sample sizes between the different geographical sub‐regions can reduce the power of detecting the total amount of local genetic differentiation, the PCA performed on the new Bulgarian samples after quality checks overall suggested a relatively homogeneous group from a genetic viewpoint (Figure S2). Accordingly, both ANOVA on PC1 (*p*‐value: 0.726) and PC2 (*p*‐value: 0.0945), performed to test if the average coordinates were significantly different among geographical regions, as well as the AMOVA analysis used to verify if F_ST_ among Bulgarian sub‐regions was significantly different from zero (*p*‐value: 0.4637), returned not significant results. For these reasons, the Bulgarian samples under study were considered as a single population group for subsequent analyzes.

**FIGURE 1:**
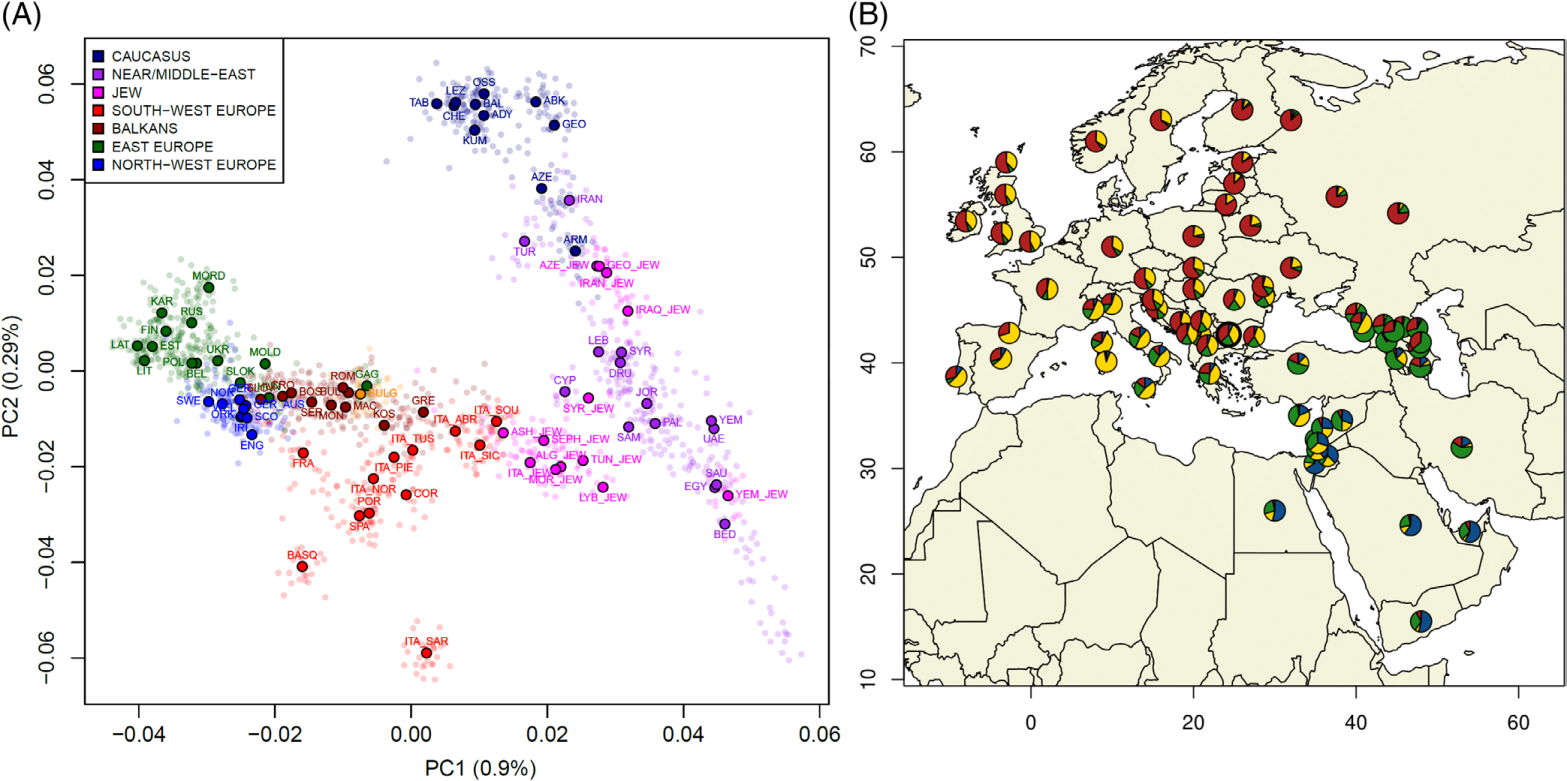
Genetic structuring patterns observed for the modern Euro‐Mediterranean population dataset. (A) Scatterplot of the first vs. second principal components. Modern individuals (faded dots) and median population coordinates (enlarged black‐bordered circles) are color‐coded based on geographic or ethnic affiliation, as in the legend at the top‐left. The new Bulgarian samples from the present study are highlighted in gold. (B) Average proportions of ancestral components inferred from ADMIXTURE at the best value of *K* = 4. The relative proportions of inferred genetic components (yellow: Sardinian/South‐Western European, red: North‐Eastern European, green: Caucasian, blue: Middle‐Eastern) are summarized by corresponding pie‐charts at population level. The Bulgarian population newly‐analyzed in the present study is highlighted in bold.

**FIGURE 2:**
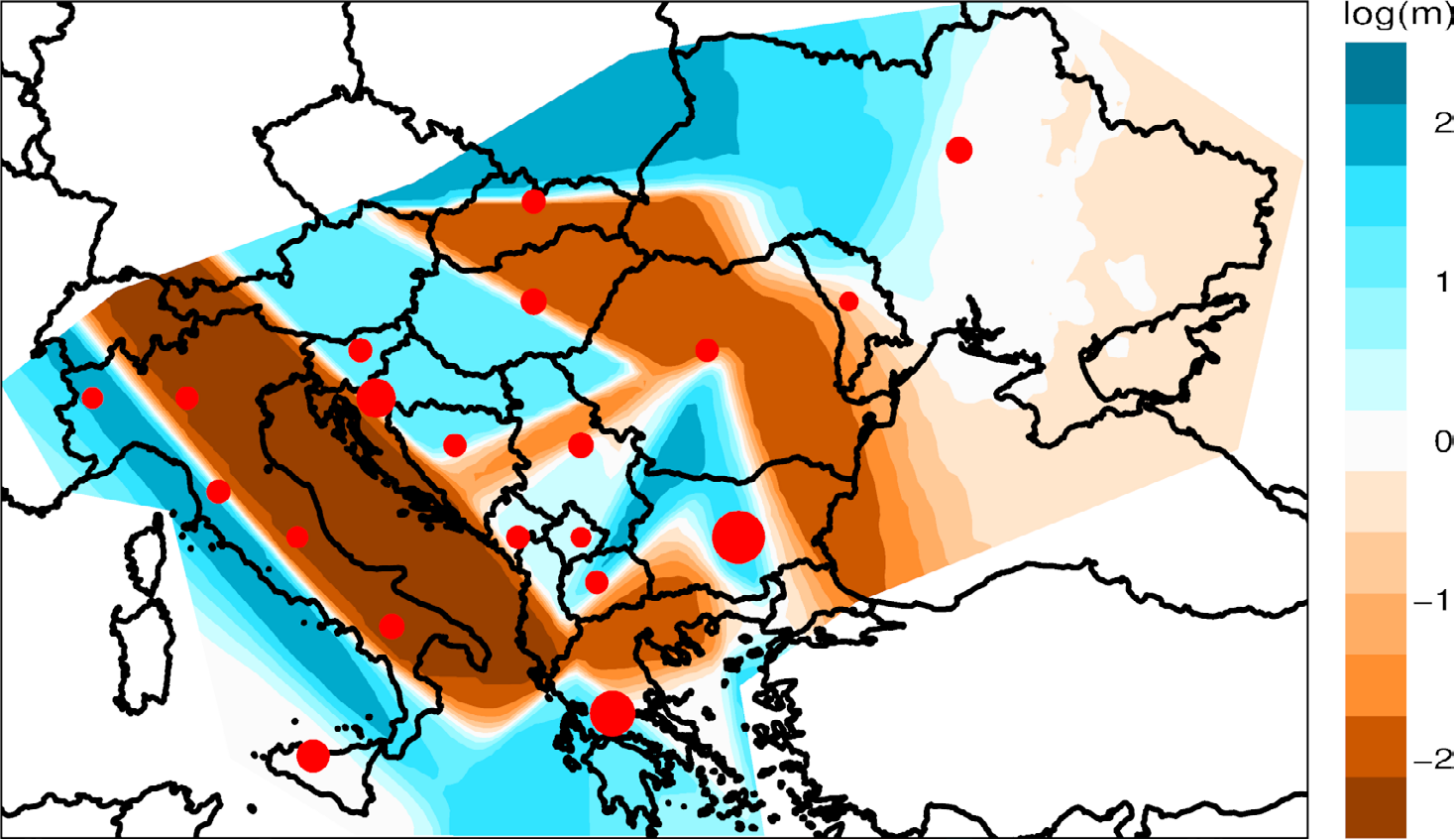
Estimated effective migration surface analysis for Bulgarian and surrounding populations, showing the posterior probabilities of the effective migration rates estimated by EEMS. Blue colored regions indicate areas of high connectivity, while orange regions indicate areas of lower gene flow. Black circles show the geolocations of populations included in the analysis, aligned to the nearest node on the grid, with circle size scaled to the number of samples included in each population point.

The position of the newly‐analyzed Bulgarians in the context of the Euro‐Mediterranean genetic landscape was then explored with a PCA analysis performed on the pruned modern extended dataset consisting of 1571 individuals from 84 populations. Additionally, we used a UPGMA tree to visualize F_ST_ pairwise genetic distances between populations. At a macro‐geographic perspective, both PCA and F_ST_ results (Figures 1A and S3) recapitulate the main West‐Eurasian structuring patterns by distinguishing the East‐to‐West gradient extending between European populations on one hand, and the genetic variation stretching from Near Eastern to Caucasian groups on the other hand. In this context, populations from the Balkan Peninsula exhibit relatively low autosomal genetic distances from one another. Accordingly, our newly‐analyzed Bulgarian samples fall within the genetic variability of the other Balkan groups, appearing closely related to Romanians, North Macedonians, and Greeks, and then clustering with the South‐Slavic populations from the Western Balkans. Consistent with these results, the Estimated Effective Migration Surface obtained with the EEMS method (Figure 2) indeed highlighted an area of high genetic similarity that covers Bulgaria and links it to southern Balkan groups. At the same time, the EEMS analysis also showed some discontinuities, with the Adriatic Sea and the Carpathian Mountains acting as areas with lower‐than‐average genomic exchanges, while corridors of higher effective migration connect South Italy to Greece and the Aegean Sea on one hand, and Eastern Europe with Slovenia, Hungary, and Northern Balkans on the other hand. To formally model the genetic components of present‐day populations, we used the clustering algorithm implemented in ADMIXTURE (Figure S4). At the best predictive value of *K* = 4 (Figure S5), Bulgarian samples, similarly to their geographically neighboring populations, display larger proportions of two major ancestry components, respectively a “Sardinian/South‐Western European” (36%) and a “North‐Eastern European” (37%) component, with the remaining part of their ancestry being accounted for by a “Caucasian‐like” (20%) component and, to a lesser extent, by a “Middle Eastern‐like” (7%) one (Figure 1B).

**FIGURE 3:**
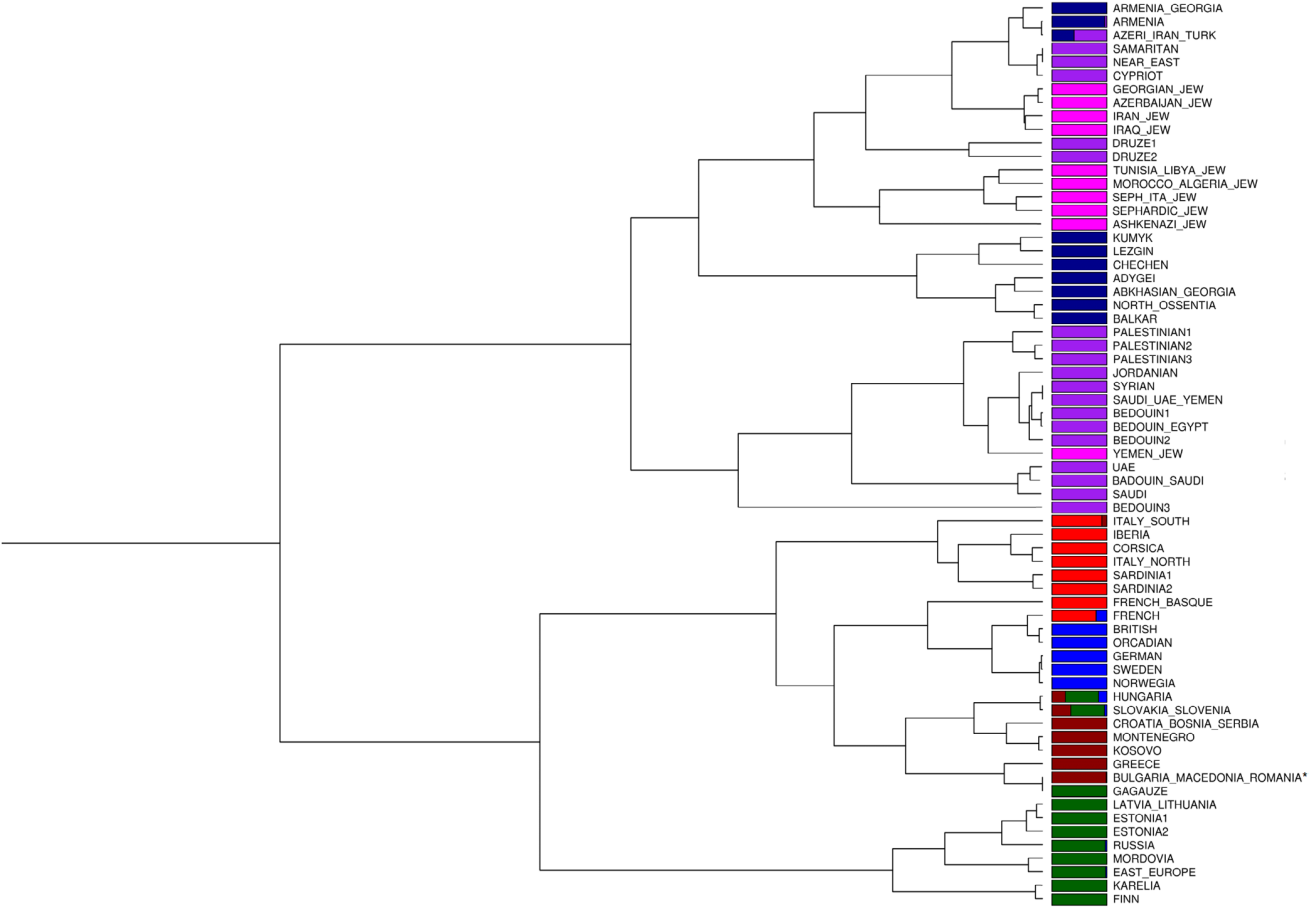
Schematic representation of the tree obtained by FineSTRUCTURE showing the 67 inferred genetic clusters. The composition of each cluster is represented by a barplot color‐coded according to the ancestry region/group of origin of the corresponding samples (dark‐blue: Caucasus; purple: Near/Middle‐East; magenta: Jews, red: South‐West Europe; dark‐red: Balkans; blue: North‐West Europe; dark‐green: Eastern Europe) as detailed in Table S4.

To describe genetic substructures and evaluate relationships among populations at a finer scale, we then used the CHROMOPAINTER/fineSTRUCTURE haplotype‐based approach, a method that allows to infer clusters of genetically homogeneous individuals by reconstructing each analyzed sample as a mosaic of genomic fragments inherited from the other “donor” individuals. On the whole, the basal structure reconstructed by fineSTRUCTURE (Figure 3) largely mirrors the general pattern observed in genotype‐based analyzes by identifying two macro‐groups that broadly divide Caucasus and Near/Middle‐East from Europe. In particular, the Southern Caucasian populations of Azeris, Armenians, and Georgians form sister clusters with those from Cyprus and the Levant, then connecting with clusters encompassing Northern Caucasian populations (Lezgins, Chechens, Balkars, Adygei, Ossentias, Abkhasians). Another different group then gathers other Near Eastern and Middle Eastern clusters, including Palestinians, Syrians, and Jordanians along with Saudis, UAE, Yemenis, as well as Egyptians and Bedouins. Within the “European branch”, an initial division distinguishes a North‐Eastern European group, wherein clusters comprising Pole, Belarusian, Mordovian, Ukrainian, Russian, and Baltic populations are grouped together with a branch primarily including Karelian and Finnish clusters. A further group encompasses South‐Western European population clusters from Iberian Peninsula, Corsica, Northern Italy, and Sardinia, then connecting to a Southern Italian cluster. Finally, a further subdivision separates North‐Central European clusters (including Swedes, Norwegians, Orcadians, Scottish, English, Irish, Welsh, Germans, French, and French Basques) from the South‐Eastern European and Balkan area. Herein, the Bulgarian samples form a cluster alongside Romanians, North Macedonians, and some Greek individuals, thus supporting PCA and F_ST_ results. This Eastern‐Balkan cluster is then related with continental Greek and Western‐Balkan population clusters (including Kosovars, Montenegrins, Serbians, Bosnians and Croats), and subsequently with Central‐Eastern European clusters (mainly encompassing Hungarians, Slovaks and Slovenians). The complete fineSTRUCTURE dendrogram is represented in Figure S6, together with the obtained pairwise coancestry matrix (Figure S7), and the detailed composition of inferred clusters (Table S4).

Recent admixture events contributing to shape the genetic composition of analyzed populations were inferred through GLOBETROTTER, by performing two different analyzes, “full” and “non‐local” as described previously (Busby et al. 2015; Hellenthal et al. 2014; Raveane et al. 2019; Tamm et al. 2019). In particular, in the “full” analysis all European, Middle Eastern, and Caucasian clusters inferred by FineSTRUCTURE were considered as possible sources of the target Bulgarian cluster. Conversely, in the “non‐local” analysis, we excluded as donor groups all the other clusters from the same South Eastern‐European/Balkan area. In both analyzes, a “one‐date” type of admixture, indicating a single date between two sources, was detected. The event particularly involved an almost 50%–50% mixture between an East European‐like source and a Southern European/Mediterranean‐like source (Table 1), dating between 23 and 32 generations ago. In the non‐local analysis—i.e., when the other Balkan groups were excluded as donor populations, the most representative sources of admixture were represented by *EAST_EUROPE* and *SOUTH_ITALY* clusters, whereas in the full analysis they were replaced by closer groups, respectively the *SLOVAK_SLOVENIAN* and *GREEK* clusters (Table 1).

**TABLE 1:** Admixture dates inferred by GLOBETROTTER for the Bulgarian cluster.

Analysis type	Date	95% CI	Prop source1	Bestmatch source1	Bestmatch source2
Full	26.216 (1214 ce)	23.691–28.772 (1137–1289 ce)	0.50	*SLOVAK_SLOVENIAN*	*GREEK*
Non‐local	27.285 (1181 ce)	23.478–31.959 (1041–1296 ce)	0.45	*EAST_EUROPEAN*	*ITALIAN_SOUTH*

*Note:* For each of the performed analyzes, the date estimates and the 95% CI obtained from 100 bootstrap resamples are reported both in generations ago and in ce years notation (between brackets), using a generation time of 30 years for the conversion. The relative proportions and the best‐matching sources identified for each event of admixture are also indicated.

### 3.2 Ancient Contributions to the Present‐Day Genetic Variation

**FIGURE 4:**
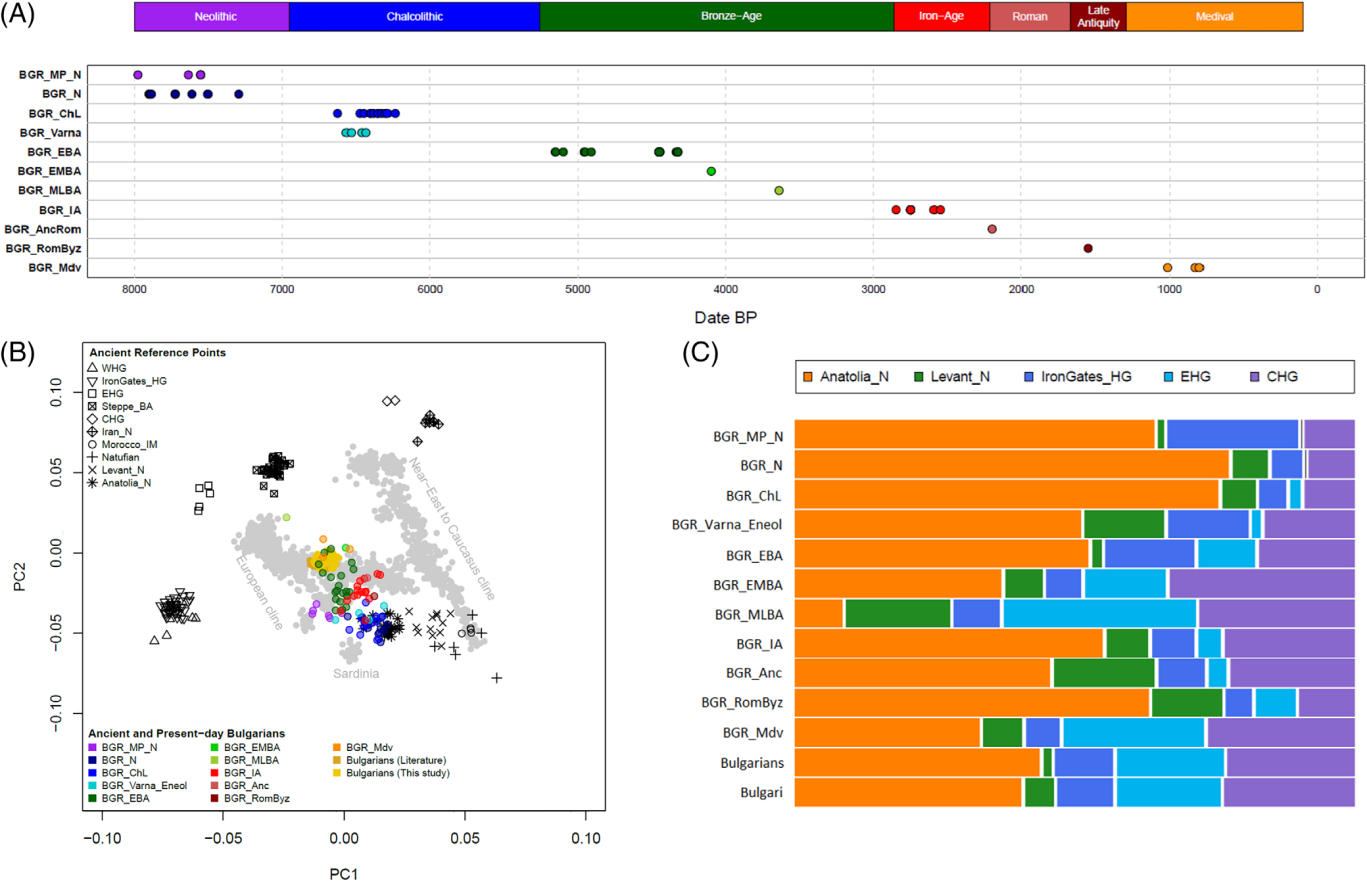
Ancestral contributions to Bulgarian populations. (A) Timeline showing the chronological distribution of considered ancient Bulgarian individuals extracted from the literature. Symbols are color‐coded based on the different period/culture. (B) Principal Component Analysis performed by projecting ancient samples onto the space defined by modern individuals. Ancient individuals from different time periods and present‐day samples from Bulgaria are symbol‐ and color‐coded as reported in the legends of the plot. (C) Mosaic plot of the ancient mixture proportions estimated using *qpAdm*. Admixture profiles have been tested using five‐way mixture models including Anatolian Neolithic, Levant Neolithic, Mesolithic Iron Gates Hunter‐Gatherers, Eastern European Hunter‐Gatherers, and Caucasian Hunter‐Gatherers as putative ancient source groups. Specific details about inferred ancestry proportions and the relative levels of significance are reported in Table S5. BGR_N: Bulgarian Neolithic; BGR_MP_N: Bulgarian Neolithic from Malak Preslavets; BGR_ChL: Bulgarian Chalcolithic; BGR_Varna: Bulgarian Eneolithic from Varna; BGR_EBA: Bulgarian Early Bronze Age; BGR_EMBA: Bulgarian Early/Middle Bronze Age; BGR_MLBA: Bulgarian Middle/Late Bronze Age; BGR_IA: Bulgarian Iron Age; BGR_Anc: Bulgarian Ancient Rome; BGR_RomByz: Bulgarian Roman Byzantine; BGR_Mdv: Bulgarian Medieval; Bulgarians: Modern Bulgarians from literature studies; Bulgari: Modern Bulgarians from the present study.

To understand how the genetic ancestry of present‐day Bulgarians relates to past population groups, we then merged the extended modern dataset with available literature data for ancient samples (Figure 4A), and performed a PCA analysis by projecting ancient individuals onto the PCs estimated from present‐day populations. Furthermore, to formally quantify the proportions of ancestral sources contributing to the modifications in Bulgarian genetic ancestry through time, we used the *qpAdm* software to model the admixture profile of ‘target’ groups as a combination of ‘ancient sources’, given a specific set of ‘outgroups’.

In accordance with previous results (Mathieson et al. 2018), our analysis revealed that all Neolithic and Chalcolithic samples from present‐day Bulgaria project closely to Anatolian Neolithic farmers in the PCA plot (Figure 4B). Accordingly, Bulgarian Neolithic (BGR_N) and Bulgarian Chalcolithic (BGR_ChL) groups can be modeled in *qpAdm* as having almost all of their ancestry (~80%–90%) tracing to Anatolian_N. A partial exception is represented by Neolithic Bulgarian samples from Malak Preslavets (BGR_MP_N), who consistently exhibit a higher amount (~20%) of hunter‐gatherer‐related ancestry (Figure 4C, Table S5). Compared to Copper Age individuals, during the Bronze Age, the ancient Bulgarian samples (BGR_EBA/EMBA) demonstrate a progressive shift in the PCA toward Yamnaya Steppe ancestry, paralleled in *qpAdm* by increasing proportions of EHG‐ and CHG‐related ancestries, that during the Middle and Late Bronze Age (BGR_MLBA) comprehensively reached ~63% (Figure 4C, Table S5). In fact, the Steppe ancestry itself has been broadly described as a mixture between an Eastern European Hunter‐Gatherers (EHG)‐related ancestry and an ancestry related to the Hunter‐Gatherers of the Caucasus (CHG) (Jones et al. 2015; Lazaridis et al. 2016). From the Iron Age (BGR_IA) and during the Roman period (BGR_Anc and BGR_RomByz) a higher contribution from genetic ancestries related to Anatolian and Levantine populations could be detected in ancient Bulgarian samples. These components instead decreased in Medieval times, with Medieval Bulgarian samples (BGR_Med) showing increasing proportions of Eastern‐European Steppe‐related ancestries compared to the pre‐medieval Byzantine population, and exhibiting a genetic profile that began to approximate the genetics of the modern Bulgarian population (Figure 4C, Table S5). When we modeled present‐day Bulgarians including a local Balkan Iron Age‐related ancestry, an Anatolian Roman/Byzantine‐related ancestry, and a Medieval Eastern European‐related ancestry, by accounting also for a possible Anatolian Ottoman‐related ancestry (see Materials and Method for further details), a major contribution from Medieval Central/Eastern European individuals with an ancestry genetically similar to the modern Eastern European Slavic‐speaking populations was observed (Table S6).

## 4 Discussion

Thanks to its unique geographic position, Bulgaria is undoubtedly known to have played a fundamental key role during all the migration processes that interested the continent through time. Up to now, the genetic variability of the country has been deeply investigated using uniparental genetic markers (Karachanak et al. 2012, 2013; Karachanak‐Yankova et al. 2015), whereas genome‐wide autosomal‐based surveys mainly consisted of wider‐range analyzes on Europe and the whole Balkan Peninsula (Hellenthal et al. 2014; Kovacevic et al. 2014; Kushniarevich et al. 2015; Ralph and Coop 2013). In this study, we attempted to focus more deeply on the patterns of autosomal genetic variation specifically observed in present‐day Bulgarians, by taking advantage of new genomic data generated from a highly representative set of samples. By comparing them with a comprehensive collection of modern and ancient populations from Europe and the Mediterranean, we aimed at recapitulating the main patterns of population variability and the major events that shaped the genetic history and the present‐day genetic landscape of Bulgaria.

Overall, our results agree with previous findings, indicating the absence of genetic sub‐structures within the Bulgarian population (Karachanak et al. 2012, 2013), also supporting the evidence of a substantial genetic homogeneity within the Balkan Peninsula (Kovacevic et al. 2014). Both genotype‐ and haplotype‐based analyzes (Figures 1A, 3, and S3) indeed place Bulgarian samples within the South‐Eastern European variability of Balkan populations, with the highest patterns of genetic similarity being observed with North Macedonians and Romanians, and then with continental Greece and the other South‐Slavic populations of the Western Balkans. The observed genetic affinity of Bulgaria with both Slavic and non‐Slavic neighboring groups aligns with previous studies (Kushniarevich et al. 2015), suggesting extensive gene flows between countries despite linguistic differences.

The modern Bulgarian gene pool particularly showed intermediate characteristics between the Mediterranean and the Eastern European ancestry compositions, reflecting the bridging position occupied by the Balkans within the Euro‐Mediterranean context. According to the best predictive scenario featured by ADMIXTURE (Figure S5), the Balkan countries indeed exhibit similar ancestry proportions, characterized by two major genetic components that are present at different frequencies throughout Europe (Figure 1B). The first genetic component is considerably represented among Mediterranean and Southern European populations, reaching a peak (~90%) in Sardinia. The second genetic component is instead more frequent in the ancestry profiles of modern Eastern European populations, being also evident in the Balkans and showing a decreasing gradient moving south toward present‐day Greek and Aegean island groups. Previous population genetics studies suggested the presence of a shared Mediterranean genetic continuity extending from Southern Italy to Cyprus and involving Crete and the Aegean islands, where Mediterranean Greek populations appeared genetically related to Southern Italians (Sarno et al. 2017 and Figure 2). This evidence has been previously interpreted as the result of ancient genetic links possibly tracing to prehistorical migrations that occurred during the Neolithic and the Bronze Age, where the Mediterranean Sea served as a preferential crossroad (Raveane et al. 2019; Sarno et al. 2017). In continental Greece and the Balkans, these signatures may have been further modified by historical events, presumptively involving contributions from continental Europe (Sarno et al. 2017).

Our GLOBETROTTER results showed admixture events for the Bulgarian population cluster that involved Southern European (Greece or Southern Italian) and Eastern European‐related sources dating between 23 and 32 generations ago (Table 1). These time estimates, corresponding to around 1000–1300 by considering a generation time of 30 years, roughly overlap with the period of Medieval Bulgarian history extending from the Byzantine conquest and preceding the Ottoman rule of Bulgaria, that followed the migrations of Slavs and Proto‐Bulgarians after the Roman era. Since Globetrotter estimates should provide upper bounds on the dates of migrations (Hellenthal et al. 2014; Leslie et al. 2015), these results suggest the impact that these events may have had on the formation of the genetic composition of present‐day populations. Accordingly, previous studies that examined the recent genetic history of Europe revealed a high number of segments shared identical by descent (IBD) among modern South‐Eastern Europeans dating at approximately 1000–2000 years before present (YBP) (Ralph and Coop 2013). Similarly, another study on modern populations inferred multiway admixture events occurring around the same period (1000–1600 YBP) for an Eastern European population cluster centered around Bulgaria (Hellenthal et al. 2014), particularly suggesting that population movements occurring during a period that encompasses the timeframe of the Slavic migrations could have played a role in contributing to the genetic composition of current Balkan populations.

In recent years, the possibility to retrieve DNA information also from ancient samples has increasingly refined our understanding of the ancient processes that shaped present‐day European genetic variation. In particular, besides the genetic heritage of Mesolithic European hunter‐gatherers, the analysis of both modern and ancient data suggested the major impact of genetic components related to the expansions of Anatolian Neolithic farmers and of Bronze Age Pontic‐Steppe herders (Haak et al. 2015; Lazaridis et al. 2014). Consistently with this view, the analysis of Neolithic and Chalcolithic Bulgarian samples indeed showed an almost exclusive Anatolian Neolithic ancestry composition, while from the Bronze Age there has been a gradual enrichment in Steppe contribution (Figure 4, Table S5). The ancient genetic profile of present‐day Bulgarians, reconstructed by using a distal 5‐way mixture modeling including basal Anatolian Neolithic, Levant Neolithic, Iron Gates Hunter‐Gatherers, Eastern European Hunter‐Gatherers, and Caucasian Hunter‐Gatherers putative ancestral sources, exhibits similar proportions of Anatolian Neolithic (~44%) and Steppe‐related (~43%) components, with ~10% of Mesolithic heritage (Figure 4C, Table S5).

After the Iron Age, the genetic composition of people inhabiting the Balkan region was suggested to have been influenced by additional migrations, with Balkan samples from the 1st millennium ce indeed revealing a higher ancestry heterogeneity compared to previous groups (Olalde et al. 2023). In particular, a considerable influx from Near Eastern‐related ancestries was documented in the Balkans during the period of Roman rule, with various Croatian and Serbian samples dated approximately between 1 and 300 ce (CroatiaSerbia_RomanAnatolian) that were modeled as deriving almost completely their ancestry from Roman/Byzantine groups of Western Anatolia and Northern Levant (Olalde et al. 2023). Then, further influxes were shown in the Balkans after the decline of the Western Roman Empire, with (i) the presence of a North/Central European‐related ancestry in 250–550 ce ancient Balkan samples possibly reflecting Barbarian migrations, and (ii) a large‐scale influx of individuals genetically related to modern Slavic‐speaking populations after ~700 ce. In particular, even if only three Bulgarian Medieval samples (dating ~ 900–1200 ce) were available in such a dataset, they displayed ~40%–60% of ancestry related to these Early Medieval individuals from Central/Eastern Europe (CEE_EarlyMedieval) clustering with present‐day Slavic‐speaking groups. Importantly, unlike the earlier North/Central European influx, this later Eastern‐European‐related source was proposed to have left a significant imprint also on the genetic profile of modern Balkan populations (Olalde et al. 2023).

When we tried to model the ancestry of present‐day Bulgarians as deriving entirely from local Iron‐Age populations, our findings indeed align with these previous results in rejecting the hypothesis of a genetic continuity since prior to the Roman period in the Bulgaria region. Instead, the more recent ancestry of modern Bulgarians revealed less than 12%–15% of ancestry tracing to Bulgarian Iron‐Age individuals (Bulgaria_IA), with a contribution of ~22% from a Roman/Byzantine Western Anatolian related ancestry and a significant percentage of ancestry (~56%) from Medieval Central/Eastern European individuals with an ancestry genetically similar to modern Eastern European Slavic‐speaking populations (Table S6). Instead, the genetic impact on the Bulgarian population of the Ottoman conquest appeared to be lower (~8.5%, Table S6), possibly due to the major role played by religion in the political, social, and cultural complexity of the Empire.

Overall, while these results accompany the admixture and time estimates obtained by the modern‐based analyzes in suggesting the impact that population processes occurred in the last 1000–2000 years may have had in shaping the present‐day genetic variability of Bulgaria, the future availability of additional ancient Bulgarian individuals, particularly covering the transect from the Roman period to the Medieval times, may surely help to further evaluate the participation of these historical phases in the cultural and temporal complexity of Bulgarian history. At the same time, future studies on modern Bulgarian samples focusing also on the analysis of exomic and/or whole‐genome sequencing data promise to complement the existing research by providing a comprehensive overview on the evolutionary history of this population, adding further insights into the impact of the recent demographic history on the observed genomic variation and the possible functional implications.

## Author Contributions

**Stefania Sarno:** data curation (equal), formal analysis (lead), investigation (equal), writing – original draft (lead), writing – review and editing (lead). **Fedora Piccini:** formal analysis (equal). **Paolo Abondio:** formal analysis (equal). **Elisabetta Cilli:** investigation (equal). **Elena M. Kuyumdjian:** investigation (equal). **Nedko A. Dimitrov:** investigation (equal), writing – review and editing (equal). **Chavdar D. Dilov:** investigation (equal). **Sara De Fanti:** investigation (equal). **Graziella Ciani:** investigation (equal). **Davide Gentilini:** investigation (equal). **Alessio Boattini:** formal analysis (equal), investigation (equal), writing – review and editing (equal). **Marco Sazzini:** formal analysis (equal), investigation (equal), writing – review and editing (equal). **Davide Pettener:** conceptualization (lead), resources (lead), supervision (lead). **Donata Luiselli:** conceptualization (lead), resources (lead), supervision (lead), writing – original draft (equal), writing – review and editing (lead).

## Conflicts of Interest

The authors declare no conflicts of interest.

## Supporting information


**Figure S1.** Sampling map showing the approximate geographic location of the newly collected samples color‐coded according to the different sub‐regions/population groups: North‐West Bulgaria (green), Central‐West Bulgaria (yellow), Central Bulgaria (blue), Rhodope Mountains (cyan), Thracian valley (orange), Vayovtsi ethnic‐group (magenta), Kapantsi ethnic‐group (gray). Dark‐filled points specifically highlights the samples that were taken into account in the final analyzes after genotyping quality checks (QC), while shaded colors refer to all the other collected samples.

**Figure S2.** Principal component analysis performed on the 38 Bulgarian individuals from seven different sampling areas newly‐analyzed in the present‐study after quality checks. Individuals samples (faded dots) and median population coordinates (enlarged black‐bordered and labeled circles) are color‐coded based on the sampling location/population group: North‐West Bulgaria (dark red), Central‐West Bulgaria (light green), Central Bulgaria (blue), Rhodope mountains (magenta), Thracian valley (cyan), Vayovtsi ethnic‐group (gold), Kapantsi ethnic group (dark green).

**Figure S3.** Dendrogram plot based on pairwise F_ST_ genetic distances between the Euro‐Mediterranean populations of the modern extended dataset. The color‐code is based on geographic or ethnic affiliation: East Europe (dark‐green), North‐West Europe (blue), Balkans (dark‐red), South‐West Europe (red), Near East (purple), Jews (magenta), Caucasus (dark‐blue). The Bulgarian population newly‐analyzed in the present study is highlighted in gold.

**Figure S4.** ADMIXTURE analysis performed on the modern extended dataset. At any *K*, from 2 (top) through 10 (bottom), each individual is represented by a vertical (100%) column of genetic component probabilities, colored according to the *K* reconstructed ancestral components. Individuals are grouped and labeled at population level.

**Figure S5.** Boxplot of cross‐validation (CV) errors for the ADMIXTURE runs in 10 replicates at *K* from 2 to 10. The best predictive accuracy (i.e., the lowest CV error) was achieved by the model testing *K* = 4 ancestral components.

**Figure S6.** FineSTRUCTURE clustering tree of all samples. Cluster labels refer to the population name and to the number of individuals from each population. The cluster containing the new Bulgarian samples analyzed in the present study is highlighted in red. The correspondence between the cluster composition and the given cluster name is reported in Table S4.

**Figure S7.** Heatmap coancestry matrix of average chunk‐length distribution generated by ChromoPainter. Individuals on the *x*‐ and *y*‐axes are sorted according to the fineSTRUCTURE clustering tree and color‐coded as in the legend at the bottom of the plot. The scale on the right of the plot shows lower (white) to higher (black) amount of shared genetic chunks between couples of individuals.

**Table S1.** List of Bulgarian samples from 7 different sampling areas newly‐analyzed in the present study.

**Table S2.** List of 1571 present‐day samples from 84 Euro‐Mediterranean populations included in the modern extended dataset.

**Table S3.** List of 522 ancient individuals considered for the comparison with modern populations.

**Table S4.** Composition of the genetic clusters inferred by ChromoPainter/FineSTRUCTURE.

**Table S5.** Results of the distal 5‐way admixture modeling of ancient and present‐day Bulgarian groups tested with *qpAdmix*.

**Table S6.** Results of the proximate *qpAdm* models tested for present‐day Bulgarian populations. One‐way mixture models were preliminary performed to test population continuity in the Bulgarian populations since prior to the Roman period. Then, present‐day Bulgarian groups were modeled using a 4‐way approach as suggested by Olalde et al. (2023).

## Data Availability

The genotype data for the newly‐analyzed Bulgarian samples generated during the current study are available at the Figshare repository (https://figshare.com/authors/Marco_Sazzini/6292466). All data on modern and ancient populations used for comparisons are publicly available from previous studies, see respective citations for the sources.
