# Supplementary figures and images for: The Genetic Variability of Present‐Day Bulgarians Captures Ancient and Recent Ancestral Contributions
Source: Am J Biol Anthropol. 2025 Apr 9;186(4):e70037. doi: 10.1002/ajpa.70037 (PMC11980028; doi:10.1002/ajpa.70037)

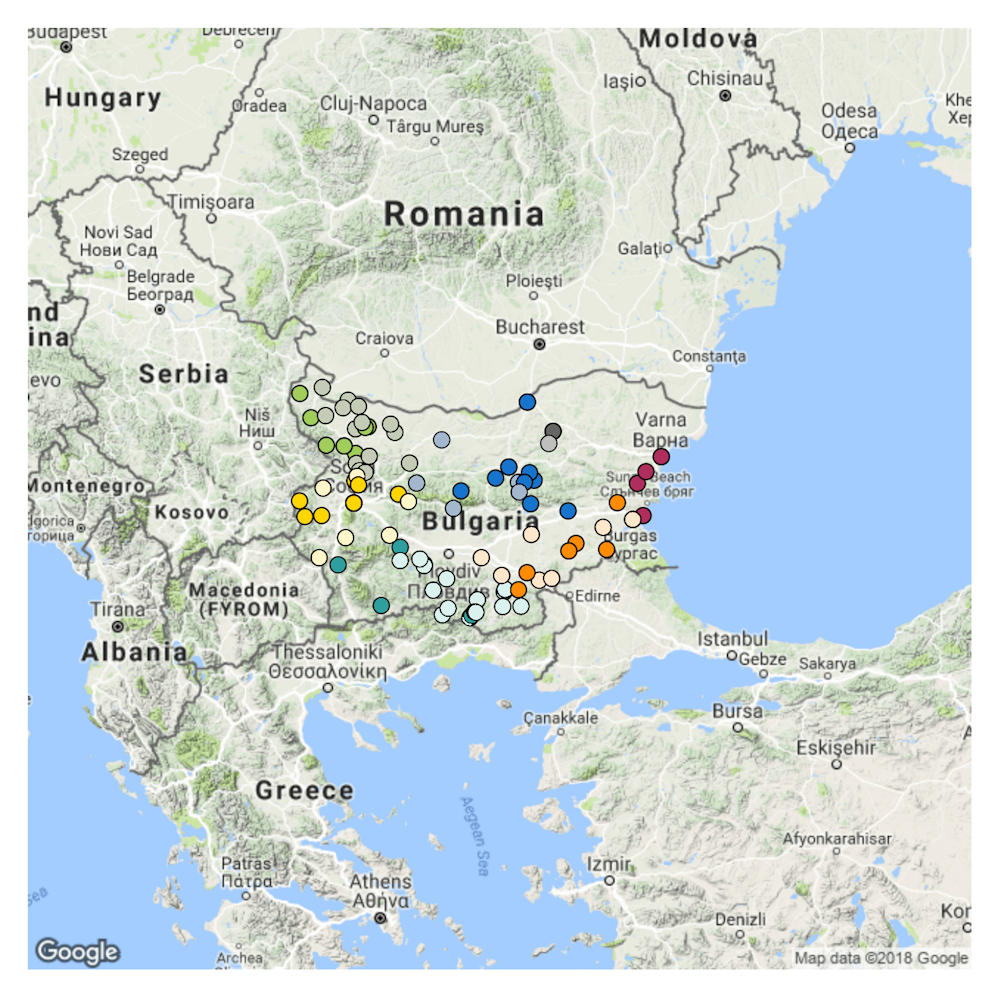

Supplement: Supplementary file 1 — Figure S1. Sampling map showing the approximate geographic location of the newly collected samples color‐coded according to the different sub‐regions/population groups: North‐West Bulgaria (green), Central‐West Bulgaria (yellow), Central Bulgaria (blue), Rhodope Mountains (cyan), Thracian valley (orange), Vayovtsi ethnic‐group (magenta), Kapantsi ethnic‐group (gray). Dark‐filled points specifically highlights the samples that were taken into account in the final analyzes after genotyping quality checks (QC), while shaded colors refer to all the other collected samples. [file AJPA-186-e70037-s007.tiff]

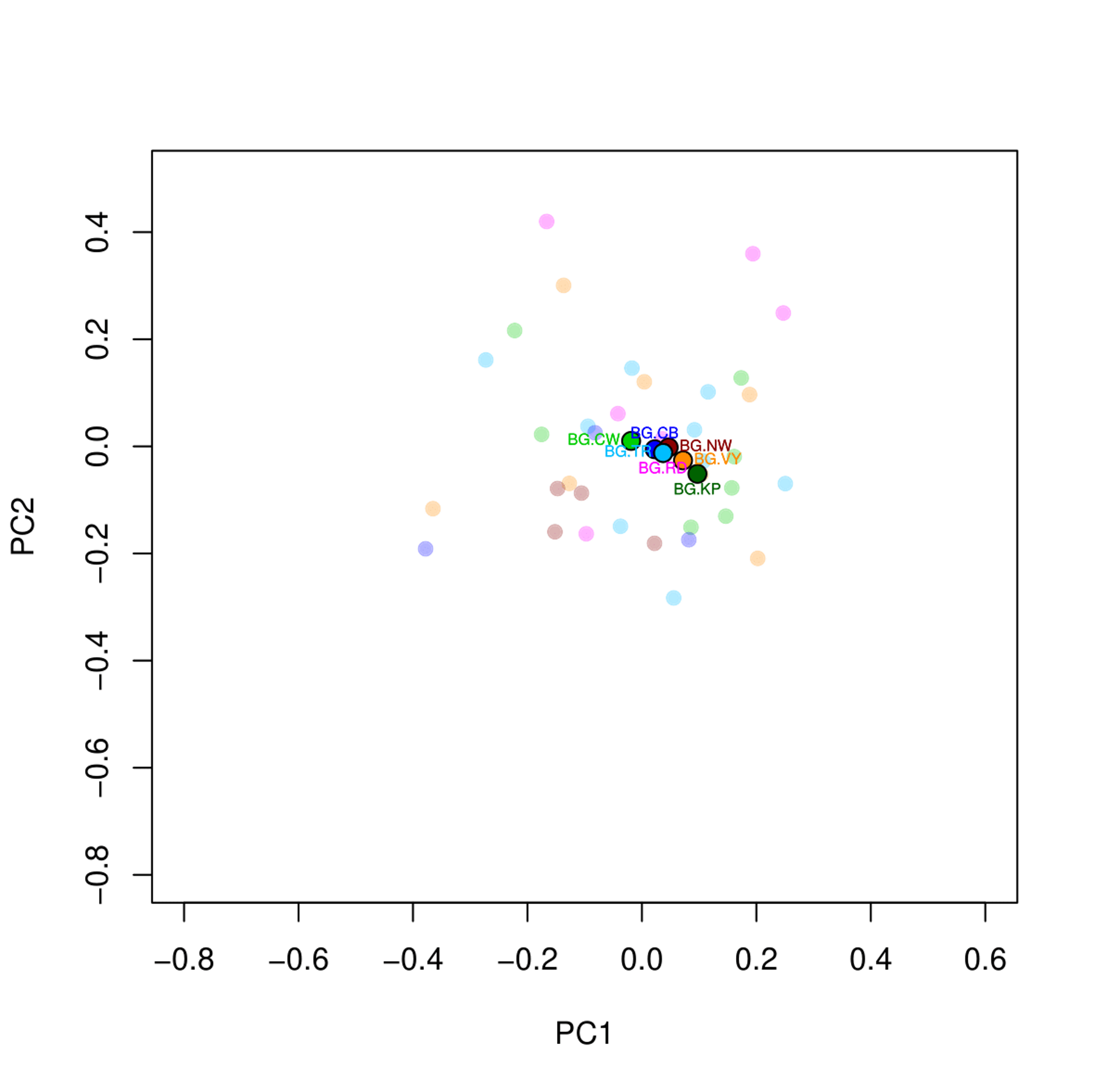

Supplement: Supplementary file 2 — Figure S2. Principal component analysis performed on the 38 Bulgarian individuals from seven different sampling areas newly‐analyzed in the present‐study after quality checks. Individuals samples (faded dots) and median population coordinates (enlarged black‐bordered and labeled circles) are color‐coded based on the sampling location/population group: North‐West Bulgaria (dark red), Central‐West Bulgaria (light green), Central Bulgaria (blue), Rhodope mountains (magenta), Thracian valley (cyan), Vayovtsi ethnic‐group (gold), Kapantsi ethnic group (dark green). [file AJPA-186-e70037-s013.png]

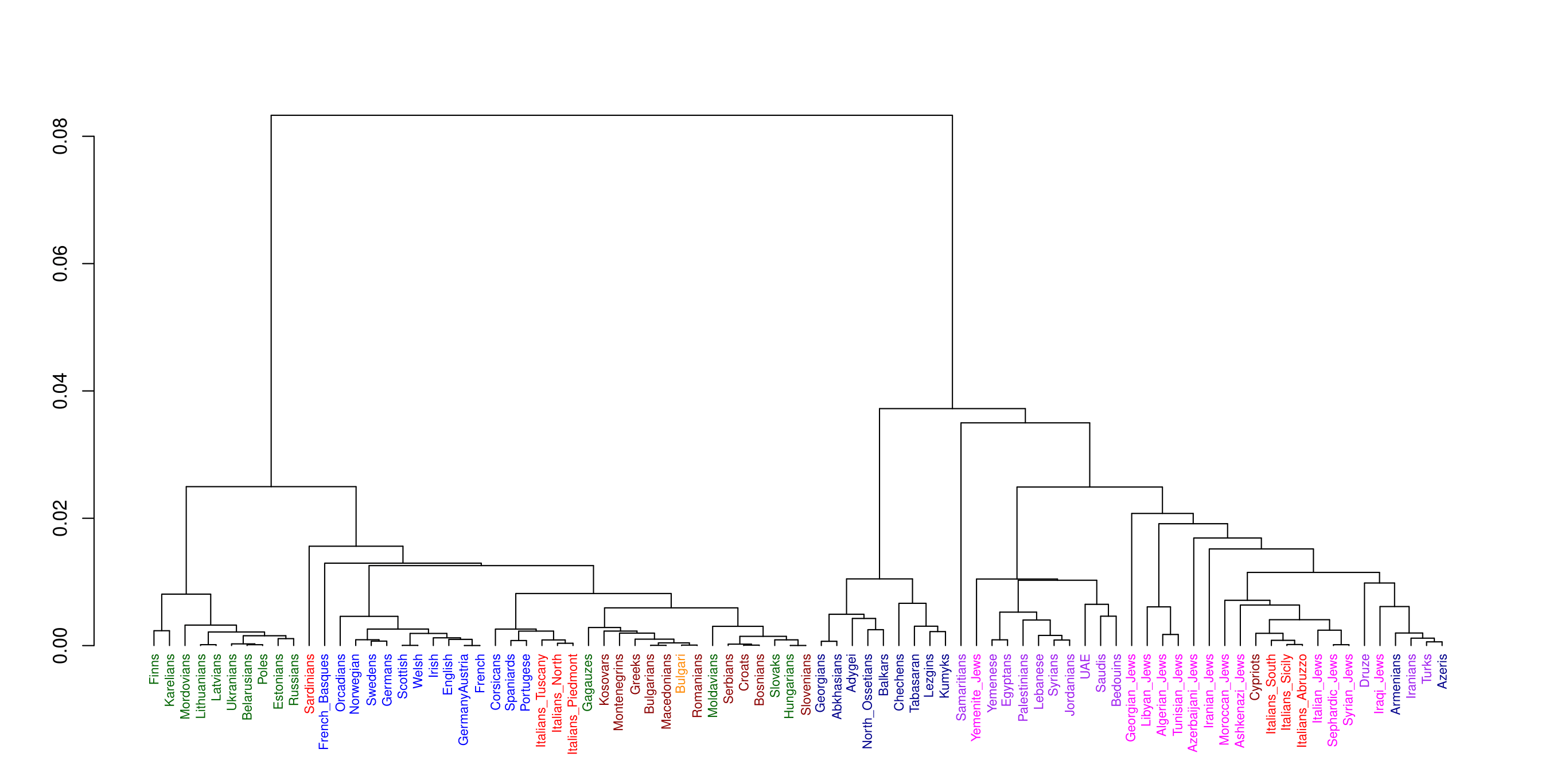

Supplement: Supplementary file 3 — Figure S3. Dendrogram plot based on pairwise FST genetic distances between the Euro‐Mediterranean populations of the modern extended dataset. The color‐code is based on geographic or ethnic affiliation: East Europe (dark‐green), North‐West Europe (blue), Balkans (dark‐red), South‐West Europe (red), Near East (purple), Jews (magenta), Caucasus (dark‐blue). The Bulgarian population newly‐analyzed in the present study is highlighted in gold. [file AJPA-186-e70037-s005.png]

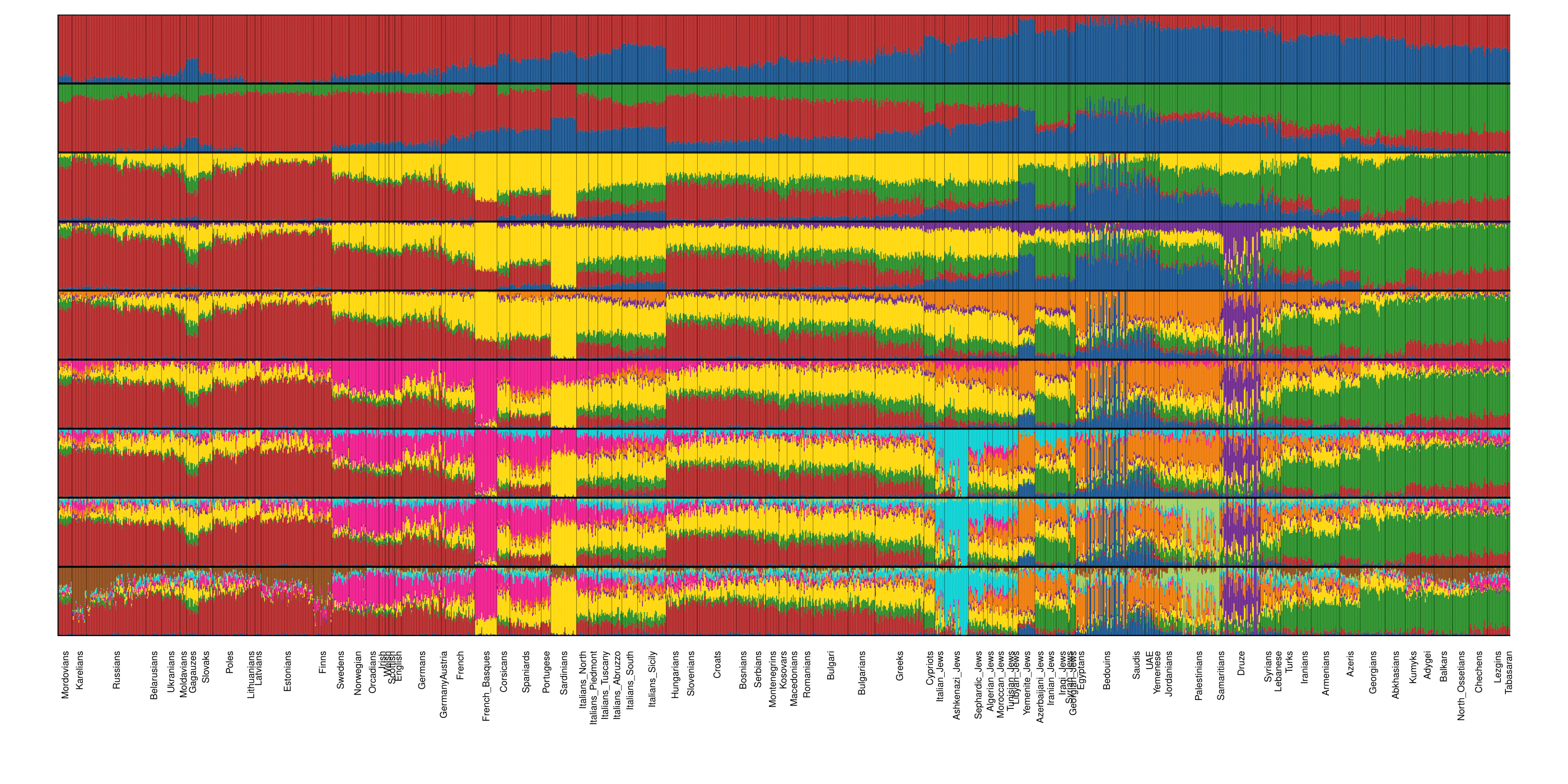

Supplement: Supplementary file 4 — Figure S4. ADMIXTURE analysis performed on the modern extended dataset. At any K, from 2 (top) through 10 (bottom), each individual is represented by a vertical (100%) column of genetic component probabilities, colored according to the K reconstructed ancestral components. Individuals are grouped and labeled at population level. [file AJPA-186-e70037-s006.png]

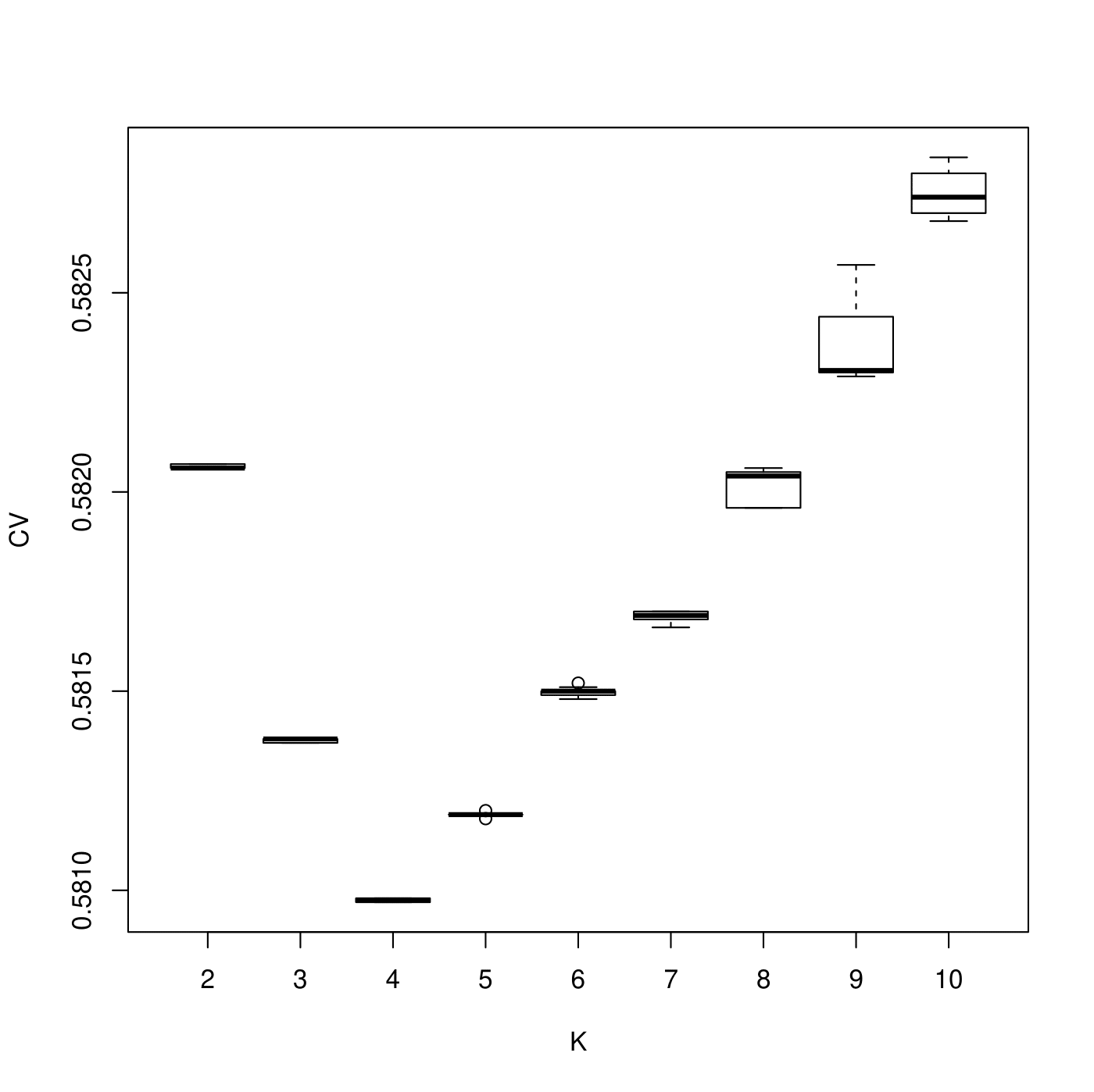

Supplement: Supplementary file 5 — Figure S5. Boxplot of cross‐validation (CV) errors for the ADMIXTURE runs in 10 replicates at K from 2 to 10. The best predictive accuracy (i.e., the lowest CV error) was achieved by the model testing K = 4 ancestral components. [file AJPA-186-e70037-s002.png]

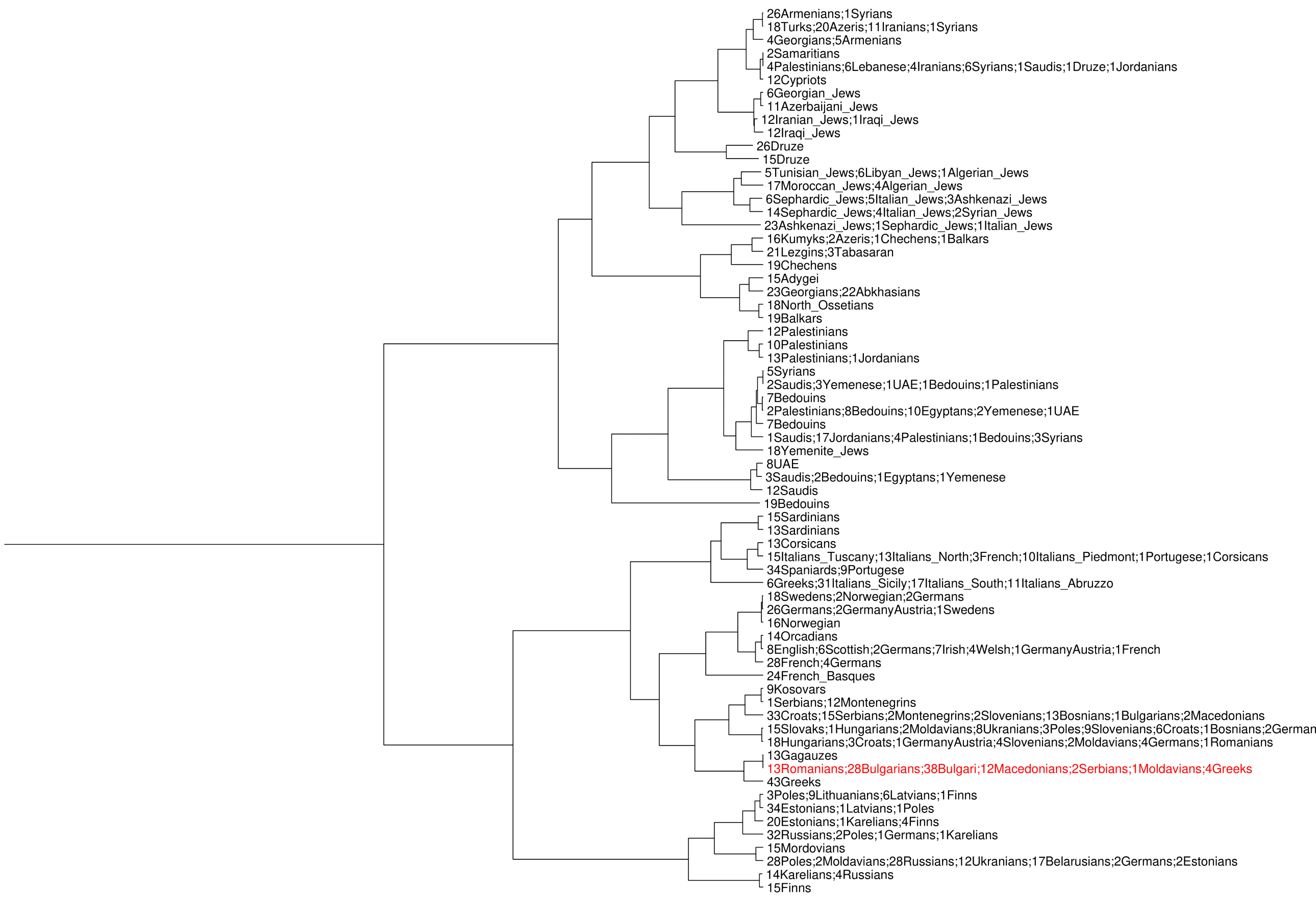

Supplement: Supplementary file 6 — Figure S6. FineSTRUCTURE clustering tree of all samples. Cluster labels refer to the population name and to the number of individuals from each population. The cluster containing the new Bulgarian samples analyzed in the present study is highlighted in red. The correspondence between the cluster composition and the given cluster name is reported in Table S4. [file AJPA-186-e70037-s011.png]

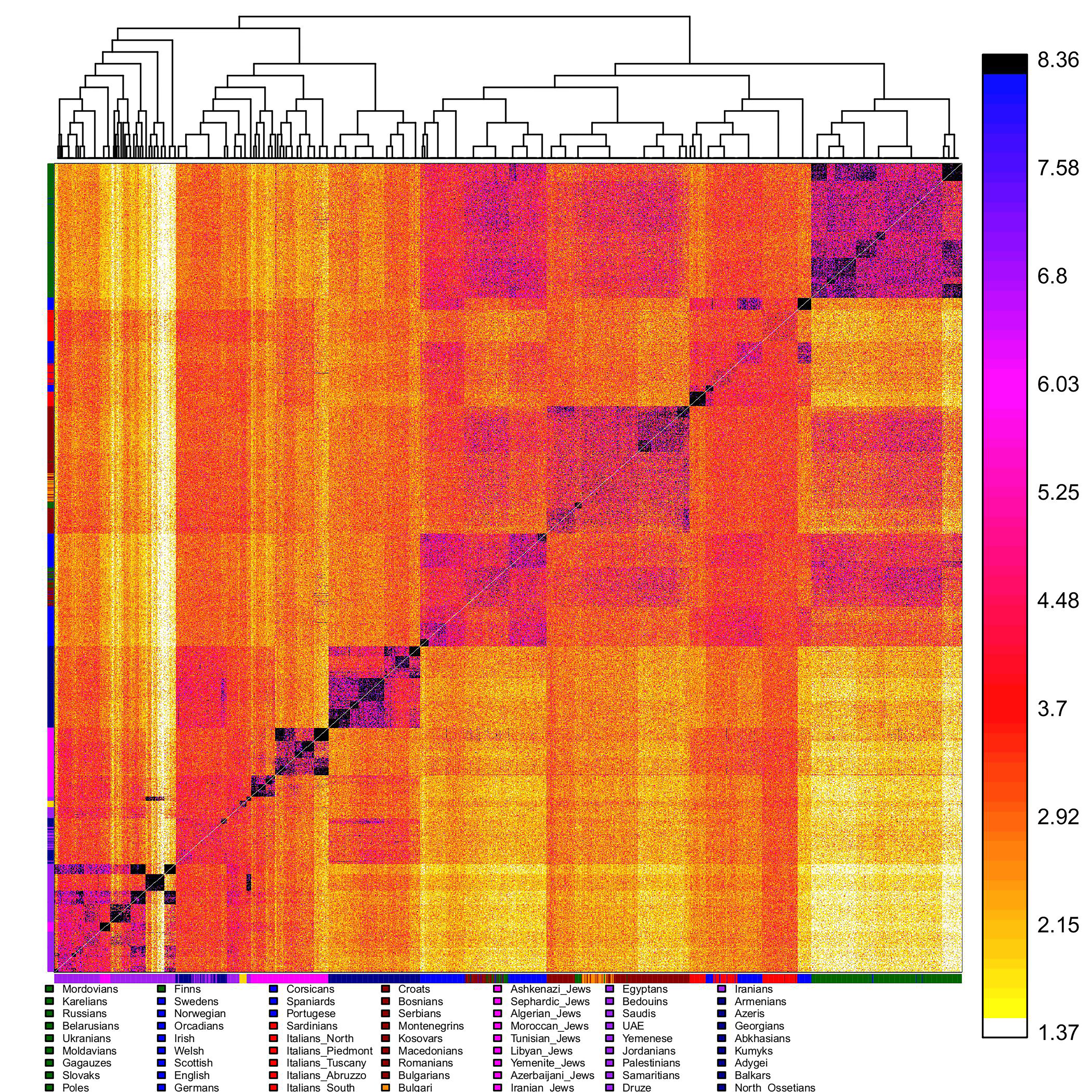

Supplement: Supplementary file 7 — Figure S7. Heatmap coancestry matrix of average chunk‐length distribution generated by ChromoPainter. Individuals on the x‐ and y‐axes are sorted according to the fineSTRUCTURE clustering tree and color‐coded as in the legend at the bottom of the plot. The scale on the right of the plot shows lower (white) to higher (black) amount of shared genetic chunks between couples of individuals. [file AJPA-186-e70037-s003.png]
